# Novel model of secreted human tau protein reveals the impact of the abnormal N-glycosylation of tau on its aggregation propensity

**DOI:** 10.1038/s41598-019-39218-x

**Published:** 2019-02-19

**Authors:** Yelena Losev, Ashim Paul, Moran Frenkel-Pinter, Malak Abu-Hussein, Isam Khalaila, Ehud Gazit, Daniel Segal

**Affiliations:** 1 Department of Molecular Microbiology and Biotechnology, School of Molecular Cell Biology and Biotechnology, Tel Aviv University, Ramat Aviv, Tel Aviv 6997801 Israel; 2 Department of Biotechnology Engineering, Ben-Gurion University of Negev, Beer Sheva, 84105 Israel; 3 Department of Materials Science and Engineering Iby and Aladar Fleischman Faculty of Engineering, Tel Aviv University, Ramat Aviv, Tel Aviv 6997801 Israel; 4 Sagol Interdisciplinary School of Neuroscience, Tel Aviv University, Ramat Aviv, Tel Aviv 6997801 Israel

## Abstract

Alzheimer’s disease (AD) is the most common neurodegenerative disorder and has no disease-modifying treatment yet. The hallmarks of AD are two amyloidogenic proteins: tau and amyloid β (Aβ). Tau undergoes several posttranslational modifications, including N-glycosylation. Tau was reported to be N-glycosylated in AD brains, but not in healthy counterparts, which may affect AD etiology. Here, we aimed to examine the effect of N-glycosylation on aggregation propensity of tau. To that end, a novel SH-SY5Y cell-based model was generated in which recombinant human tau (htau) is forced to be secreted from the cells. Secreted htau was found to localize in the secretory pathway compartments and to undergo N-glycosylation. Following N-glycan cleavage of the secreted htau, various biophysical results collectively indicated that the untreated N-glycosylated secreted htau is markedly less aggregative, contains thinner and shorter fibrils, as compared to treated de-glycosylated secreted htau. This finding shows that N-glycans attached to htau may affect its aggregation. This could help to better understand the effect of N-glycosylated htau on AD progression.

## Introduction

Alzheimer disease (AD) is a severe progressive neurodegenerative disorder and a major cause of dementia for which no disease-modifying treatment is currently available^1–3^. Although the exact molecular mechanism leading to AD is not fully understood, two hallmark lesions of the disease are extracellular senile plaques (SPs), composed of insoluble fibrils of Amyloid β peptide (Aβ_40_ and Aβ_42_) and intraneuronal deposits of neurofibrillary tangles (NFTs) composed of paired helical filaments (PHFs) of aggregated hyperphosphorylated tau protein^4,5^. Tau is an intrinsically disordered protein belonging to a family of microtubule associated proteins (MAPs), and its primary function is to stabilize microtubules^6–8^. Tau is a cytosolic protein that exists in six major isoforms and is abundant in neurons^7^.

Various post translational modifications regulate tau function, including phosphorylation, O-GlcNAcylation, N-glycosylation, isomerization, glycation, nitration, acetylation, oxidation, polyamination, sumoylation, ubiquitination and truncation^9^. Among these, normal phosphorylation is crucial for tau binding to microtubules, whereas hyperphosphorylation of tau leads to its dissociation from the microtubules, which are consequently disrupted, as well as to its subsequent aggregation and accumulation as cytotoxic NFTs^10^. Tau from human brain was found to undergo O-GlcNAcylation on Serine and Threonine residues, which is competitive with their phosphorylation. Growing evidence suggests that the interplay between these two posttranslational modifications of tau impact AD etiology^11,12^. In addition to being O-GlcNAcylated, hyperphosphorylated tau and PHF-tau, were found to be N-glycosylated in the AD brain but not in healthy brains^13,14^. The finding of N-glycosylation of tau is surprising because this protein is cytosolic whereas the N-glycosylation machinery resides in the endoplasmic reticulum (ER) and Golgi.

N-glycosylation involves attachment of oligosaccharides to the Asparagine residues of the protein. In eukaryotes, this process is co-translational, occurring in the ER and the N-glycans are processed in the Golgi compartment. The precursor sugar (Glc3Man9GlcNAc2) is attached to a consensus sequence of Asp-X-Ser/Thr by an oligosaccharyl transferase (OST) complex, followed by further processing of the sugar in the ER and Golgi^6^. N-glycosylation was found to impact stability, folding, oligomerization and solubility of the glycoproteins^15–17^. N-glycosylation of various AD related proteins including APP, BACE1 and ADAM10 was found to have a role in disease development and progression (for recent reviews see)^14,18^. Recent works suggest that there are massive alterations of global protein glycosylation in the various pathways in AD patients^19^.

Few studies have examined the abnormal N-glycosylation of the tau protein and its involvement in AD. Both composition and structure of the N-glycans on tau were found to be partially different between hyperphosphorylated tau (p-tau) and PHF-tau in the AD brain^20^. The effect of N-glycosylation on phosphorylation and dephosphorylation of tau was also examined. It was found that N-glycosylated tau from AD brains is a better *in vitro* substrate for phosphorylation by protein kinase A (PKA), compared to the deglycosylated tau^21,22^. Additionally, subjecting PHF-tau extracted from AD brains to *in vitro* deglycosylation affected PHF structure and promoted its morphological transition towards straight filaments. Moreover, on its own deglycosylation of PHF tau did not restore its microtubule polymerization activity, whereas it did so when combined with its dephosphorylation. This indicates that glycosylation of tau has no direct impact on microtubule polymerization, but rather affects maintenance of PHF structure^13^.

While most of the research on N-glycosylation of tau has focused on identification of the sugars involved and the interplay between N-glycosylation and phosphorylation on tau from AD brains, to the best of our knowledge, no studies directly examined the effect of N-glycosylation of tau on its aggregation propensity. To overcome the scarcity of samples of N-glycosylated tau from brains of AD patients, we generated a novel *ex-vivo* model, in which human tau (htau) is fused to a signal peptide motif, thereby forcing it through the secretory pathway where it can potentially undergo N-glycosylation. Comparison of the aggregation propensity of media containing secreted htau before and after enzymatic removal of N-glycans using peptide-N4-(N-acetyl-beta-glucosaminyl) asparagine amidase (PNGase-F), revealed that the former is less aggregative than the latter and comprises less dense fibrillary aggregates. This result suggests that N-glycosylation reduces the aggregation propensity of secreted htau. This cell-based model provides novel means for studying tau engendered AD pathology.

## Results

**Figure 1:**
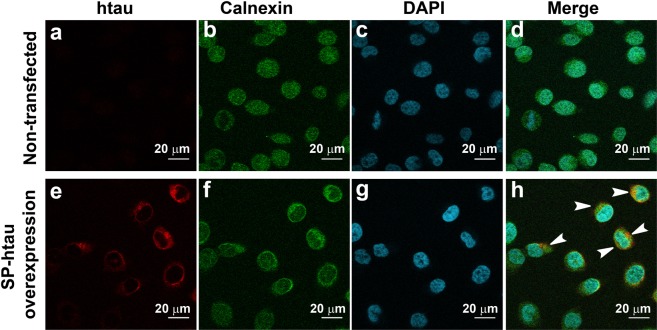
SP-htau overexpressed in SH-SY5Y cells is colocalized with Calnexin to the ER: (**a**–**d**) Non-transfected SH-SY5Y cells; (**e**–**h**) SH-SY5Y cells stably transfected with SP-htau; (**a**,**e**) htau staining using 5A6 antibody against total tau; (**b**,**f**) anti Calnexin staining; (**c**,**g**) nuclear staining using DAPI; (**d**) Merge of (**a**–**c**). (**h**) Merge of (**e**–**g**). The arrows point to the colocalization of tau and Calnexin.

**Figure 2:**
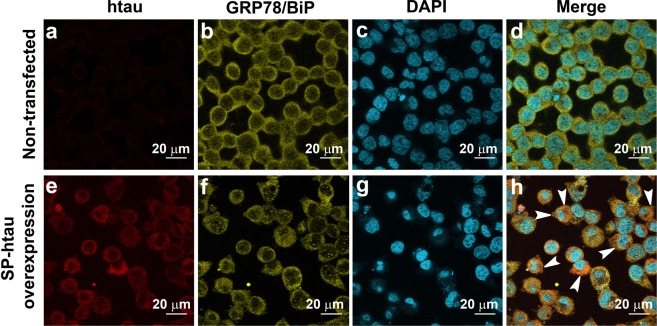
SP-htau overexpressed in SH-SY5Y cells is colocalized with GRP78/BiP to the ER: (**a**–**d**) Non-transfected SH-SY5Y cells; (**e**–**h**) SH-SY5Y cells stably transfected with SP-htau; (**a**,**e**) htau staining using 5A6 antibody against total tau; (**b**,**f**) anti GRP78/BiP staining; (**c**,**g**) nuclear staining using DAPI; (**d**) Merge of (**a**–**c**). (**h**) Merge of (**e**–**g**). The arrows point to the colocalization of tau and GRP78/BiP.

The unexpected N-glycosylation of htau reported in the brains of AD patients but not in age-matched healthy controls may impact disease etiology by affecting htau aggregation. To investigate the effect of N-glycosylation on aggregation propensity of htau, a cellular model was generated overexpressing a secreted version of htau (SP-htau), which upon passing through the secretory pathway can serve as a substrate for the N-glycosylation machinery. In order to force secretion of htau outside of the cells, a signal peptide was fused upstream to the htau sequence and the construct was stably transfected into SH-SY5Y neuroblastoma cells. First, we examined whether SP-htau in these cells passes through a secretory pathway. To that end, the transfected cells were immunostained using both 5A6, an antibody detecting total tau, and sc-11397, recognizing calnexin, an ER membrane bound chaperon^23^. Non-transfected SH-SY5Y cells displayed minimal background signal using the 5A6 antibody to total tau (Fig. 1a), probably reflecting expression of the endogenous protein^24^. In contrast, a strong 5A6 signal was evident inside the SP-htau transfected cells due to overexpression (Fig. 1e). Calnexin signal was evident in both cell types (Fig. 1b,f and Supplementary Fig. S1) and colocalization of SP-htau and calnexin signals was clearly observed in SP-htau expressing cells (Fig. 1h and Supplementary Fig. S1) but not in non-transfected cells (Fig. 1d and Supplementary Fig. S1), suggesting that SP-htau is associated with the ER. Similar colocalization was observed using an antibody towards an additional ER chaperon: glucose-regulated protein (GRP78), also referred as binding immunoglobulin protein (BiP)^25^ (Fig. 2 and Supplementary Fig. S2).

**Figure 3:**
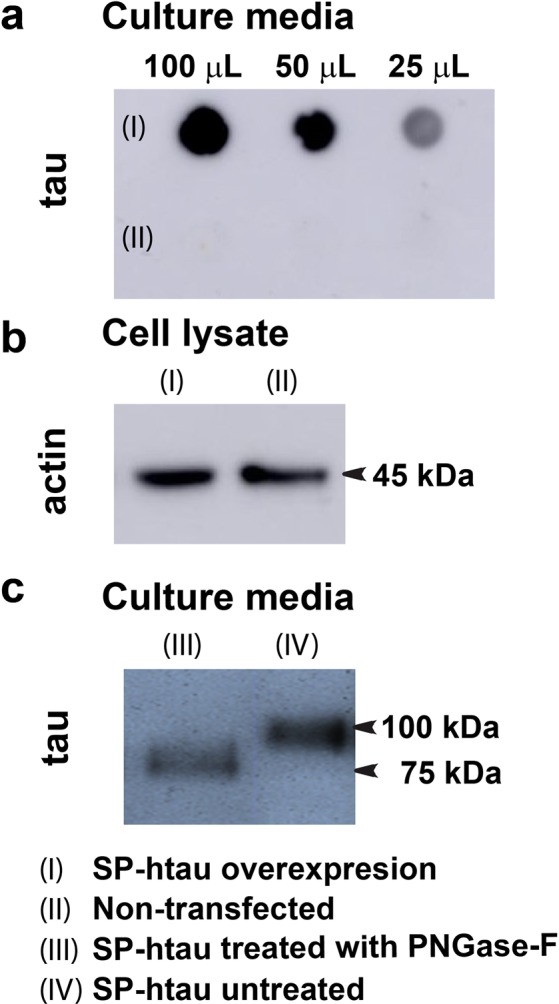
SP-htau is secreted to the medium of stably transfected SH-SY5Y cells and is N-glycosylated: (**a**) Dot blot using 5A6 antibody (against total tau) on: medium of SH-SY5Y cells overexpressing SP-htau (top panel, I) and medium of non-transfected SH-SY5Y cells (bottom panel, II). (**b**) The same cell lines used for the dot blot (left – SH-SY5Y cells overexpressing SP-htau; right – non-transfected SH-SY5Y cells), were lysed and total protein level was evaluated on Western blot, using ab8228 antibody (against β-actin). (**c**) Western blot using 5A6 antibody (against total tau) on the medium from SH-SY5Y cells overexpressing SP-htau prior to (right) and following (left) PNGase-F treatment.

To verify that the overexpressed SP-htau is further secreted from the cells in an appreciable amount, dot blot was performed on growth medium collected from culture overexpressing SP-htau and from culture of control non-transfected cells. A strong signal of tau was evident in the medium from SP-htau expressing cells (Fig. 3a,I; Supplementary Fig. S3) indicating that SP-htau was indeed secreted from these cells, whereas the medium of control non-transfected cells did not display any signal (Fig. 3a,II; Supplementary Fig. S3). As a further control we verified that the total level of proteins in both cell lines is essentially similar by lysing the cells and subjecting the samples to SDS-PAGE and Western blot analysis using an antibody towards actin (Fig. 3b; Supplementary Fig. S4). We observed an increased level of actin in the cell lysates in presence vs. absence of FBS. We noted that the total number of cells is higher in the presence of FBS than in its absence, both in non-transfected and in SP-htau overexpressing cells, indicating higher proliferation of the cells in presence of FBS (Supplementary Table S1 and Fig. S5). To exclude the possibility that SP-tau is present in cell culture media as a result of increased lysis of unhealthy cells, rather than through active secretion, we evaluated the percentage of live and dead cells in presence and absence of FBS. The difference we found between the two condition is only 7% (for non-transfected cells) and 5% (for SP-htau overexpressed cells) (Supplementary Table S1). We therefore believe that presence of SP-htau in the medium is due to active secretion from SP-htau transfected cells. We corroborated these results by fluorescent live/dead staining assay (Supplementary Fig. S5). It showed that the density of cells is higher in presence of FBS (Supplementary Fig. S5a,c) than in its absence (Supplementary Fig. S5b,d) both in non-transfected and in SP-htau overexpressing cells and there was no apparent difference in the number of dead cells. Mass spectrometry analysis indicated that htau is present in the growth medium of the SH-SY5Y cells stably transfected with SP-htau (Supplementary Table S2). The mass spectrometry data was obtained from the band corresponding to the band identified in the western blot as SP-htau.

To examine whether SP-htau secreted to the medium was N-glycosylated, peptide-N4-(N-acetyl-beta-glucosaminyl) asparagine amidase (PNGase-F) enzyme, which cleaves off N-linked oligosaccharides from glycoproteins was used^26^. Enzyme treated and untreated (control) media containing SP-htau were subjected to SDS-PAGE and Western blot analysis using 5A6 antibody towards total tau. An overt shift towards lower molecular weight was observed after treatment with PNGase-F in comparison to the untreated sample (Fig. 3c; Supplementary Fig. S6), indicating that secreted SP-htau in this model system is N-glycosylated.

To evaluate the effect of N-glycosylation on aggregation propensity of htau the aggregation profile of the media containing SP-htau before and after PNGase-F treatment was examined using ThT binding assay. Medium from non-transfected SH-SY5Y cells before and after PNGase-F treatment was used as a control.

**Figure 4:**
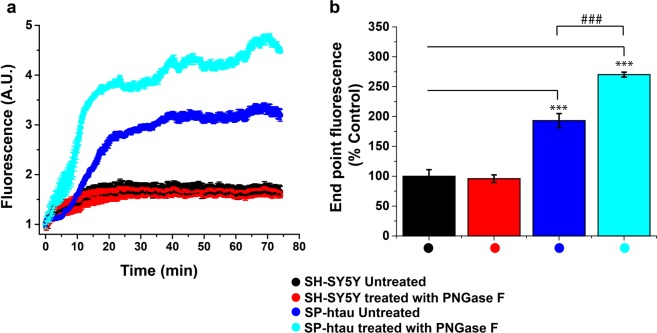
Medium containing deglycosylated SP-htau exhibits elevated aggregation: Time dependent Thioflavin-T binding kinetics of culture medium from (**a**) non-transfected SH-SY5Y cells treated (red) or untreated with PNGase-F (black), and of SH-SY5Y cells expressing SP-htau prior to (blue) and following PNGase-F treatment (cyan). (**b**) Relative ThT signal at the end point of incubation at 37 °C. Non-transfected untreated cells were set as 100%. P-values: ***p < 0.001 and ^###^p < 0.001.

In preparation for the assay, the samples were passed through centrifugal filters in an attempt to remove the cleaved off sugars, the enzyme and others salts from the solutions. ThT signal of the filtrate (Fig. 4a) indicated that the culture medium from control, non-transfected SH-SY5Y cells, exhibited very low aggregation rate, independent of PNGase-F treatment (Fig. 4, red and black curves), probably reflecting aggregation of various proteins which were secreted from the cells and remained in the filtrate. In contrast, untreated medium from SH-SY5Y cells expressing SP-htau, which contains secreted htau, aggregated at a significantly higher rate (Fig. 4a, blue curve) presumably due to the aggregation of SP-htau. Treatment of this medium (Sp-htau) with PNGase-F enhanced aggregation rate and reduced the lag phase (Fig. 4, cyan curve). These results were coroborated using ThS (Supplementary Fig. S7) instead of ThT. Taken together these observations suggest that decoration of htau with N-glycans reduces its aggregation propensity.

**Figure 5:**
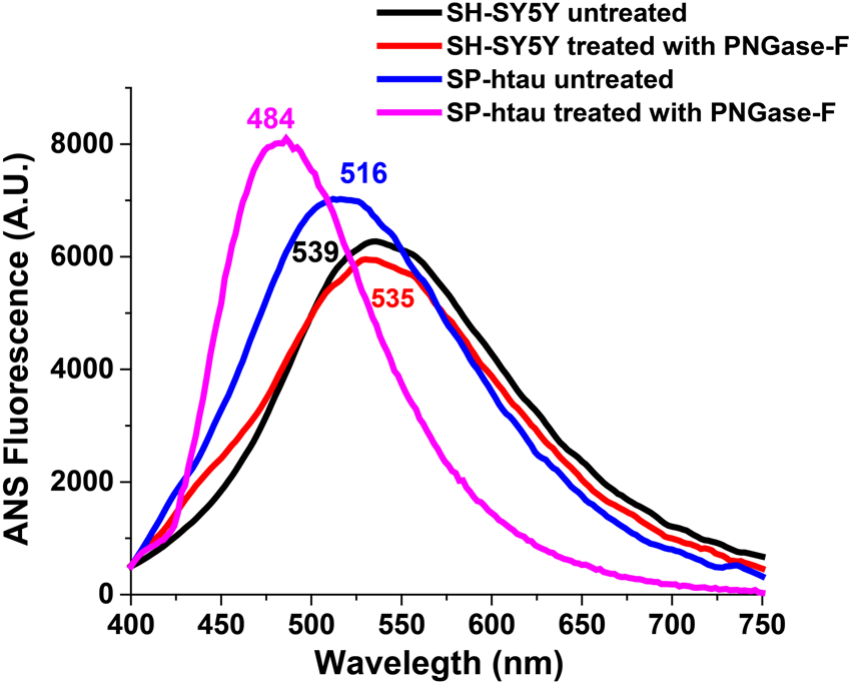
ANS binding assay of SP-htau: ANS binding assay of culture medium from non-transfected SH-SY5Y cells treated (red) or untreated with PNGase-F (black), and of SH-SY5Y cells expressing SP-htau prior to (blue) and following PNGase-F treatment (magenta). The spectra were recorded with an excitation of 380 nm and emission was from 400–750 nm.

To futher validate the amyloidogenic nature of the aggregates we  performed 1-anilinonaphthalene-8-sulfonic acid (ANS) binding assay. The binding of ANS to hydrophobic regions of amyloid assemblies is accompanied by a blue shift of the emission maximum (from ~530 nm to ~475 nm)^27^. We observed clear blue shift (to 484 nm) of emission when ANS bound to SP-htau treated with PNGase-F in comparsion to the untread SP-htau where we observed a smaller blue shift (to 516 nm). Such a shift was not apparent with either PNGase-F traeted or untreated culture media of non-transfected cells (Fig. 5). These results indicate that the deglycosylated SP-htau is more hydrophobic in nature, indicating presence of higher level of amyloids than in N-glycosylated SP-htau.

**Figure 6:**
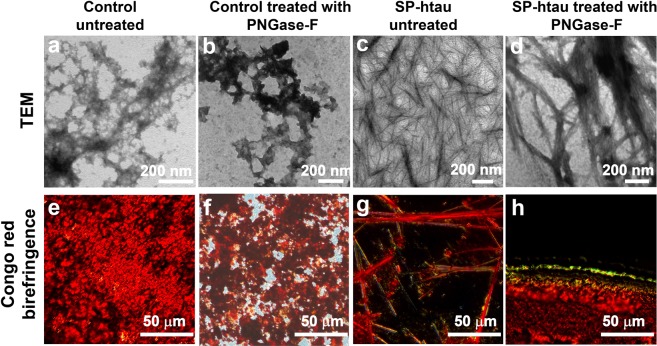
Amyloid fibrils in culture medium containing secreted SP-htau: TEM images of a sample obtained from culture medium in which non-transfected (control) SH-SY5Y cells grew, untreated (**a**) or treated (**b**) with PNGase-F; images of a sample obtained from culture medium in which SH-SY5Y cells secreting SP-htau grew, untreated (**c**) or treated (**d**) with PNGase-F. Congo red birefringence images of a sample obtained from culture medium in which non-transfected (control) SH-SY5Y cells grew, untreated (**e**) and treated (**f**) with PNGase-F and sample obtained from culture medium in which SH-SY5Y cells secreting SP-htau grew, untreated (**g)** and treated (**h**) with PNGase-F.

The structure of the aggregates in the samples from the cell culture media was exmined by transmission electron microscopy (TEM). No fibrillar assemblies were observed in medium from control non-transfected SH-SY5Y cells, whether untreated or treated with PNGase-F (Fig. 6a,b). In these media some amorphous aggregates were detected, probably originating from aggregation of various proteins that were secreted from the cells and remained in the medium after filter centrifugation. In contrast, SP-htau containing medium that was not treated with PNGase-F exhibited numerous thin fibers (Fig. 6c) and tretament with the enzyme resulted in wide bundles, which appear to be longer than the fibrils in the untreated sample (Fig. 6d). In order to confirm that the fibrils contain tau, we used immuno-gold electrom microscopy^28^. Gold labeling of the fibrils was evident in both SP-htau samples, treated and untreated with PNGase F. (Supplementary Fig. S8).

Congo red birefringence was next used to evaluate the presnece of amyloids in the various growth media. Under cross polarized light Congo red changes it color from red to green gold or apple green in presence of amyloids^29^. No birefingence was observed in samples from control non-transfected SH-SY5Y cells, whether untreated or treated with PNGase-F, implying very low level of amyloid aggregates (Fig. 6e,f). In contrast, PNGase-F untreated medium from SH-SY5Y cells expressing SP-htau, which contains secreted htau, presented some green gold birefringence (Fig. 6g) whereas PNGase-F treated medium from these cells (Fig. 6h) exhibited intense Congo red birefringence, indicating higher level of amyloids present in the sample which contains SP-htau lacking N-glycan decoration.

Collectively, the ThT, ANS, TEM, and Congo red assays are concordant indicating that medium from cells secreting N-glycosylated SP-htau is aggregative and that removal of the N-glycans enhances aggregation.

## Discussion

Glycosylation plays an important role in protein folding, stability and solubility^15,17^. The glycans render the protein more polar due to the presence of several hydroxyl groups which are hydrophilic and hence increase protein solubility and reduce the likelihood of aggregation. Glycans stabilize the protein through hydrophobic interactions between the hexose ring and the π-electrons of aromatic amino acid residues in the protein. In addition, glycans can stabilize the protein through hydrogen bonds between the hydroxyl groups of the glycans and the charged amino acids including lysine, arginine, histidine, aspartic acid and glutamic acid^30–32^. Collectively these effects of glycosylation contribute to the maintenance of the native conformation of the protein, hence ensure its normal function^16^.

For instance, we have previously reported that attaching β-O-linked glycans to the tau-derived amyloidogenic PHF6 peptide, which is commonly used as a proxy for the full length tau protein^33,34^, hindered its self-aggregation and rendered it an inhibitor of aggregation of non-glycosylated PHF6 upon co-incubation^35^. In the β-O-linked glycosylated peptide, the bulky nature of the glycans was suggested to prevent the peptide from fitting into the proximity of another PHF6 monomer, hence inhibiting self-aggregation as well as causing cross-inhibition of non-modified PHF6.

In addition to O-glycosylation, N-glycosylation plays an important role in protein folding and several reports indicate that decoration with N-glycans may reduce aggregation and enhance solubility and stability of proteins. For example, mutations in the surfactant protein A (SP-A) that impair its N-glycosylation led to formation of insoluble aggregates and decreased protein stability compared to wild type SP-A^36^. Likewise, Tunicamycin-mediated inhibition of N-glycosylation of the G protein of the vesicular stomatitis virus rendered it aggregative compared to its glycosylated version^37^. Another study showed that introduction of N-glycosylation signal sequence into amyloidogenic versions of the hen egg-white lysozyme, by substitution of glycine 49 with asparagine, resulted in an increase of their solubility^38^.

The finding that tau in the AD brain, but not in healthy brain, is N-glycosylated^13^ is unexpected since tau is a cytosolic protein, whereas the N-glycosylation machinery is associated with the ER. Several hypotheses were proposed in an attempt to explain this phenomenon: altered localization of tau, making it accessible to the N-glycosylation machinery; increased activity of oligosaccharyl transferase (OST), the complex which catalyses the addition of N-glycans to a proteins; and altered activity of cytosolic PNGase-F^18^.

Recent studies revealed that both p-tau and the PHF tangles in the AD brain contain N-glycosylated tau along with its hyperphosphorylation. Further they reported that deglycosylation of these tangles altered the PHF structure into tight filament bundles. It was suggested that glycosylation contributes to the increased stability of the PHFs structure^13^. However, the exact role of N-glycosylation in tau aggregation and in AD pathology remains to be elucidated. The novel cell-based model reported here provides a useful platform to begin interrogation of the role of N-glycosylation in tau aggregation and the underlying mechanism. Using human neuroblastoma SH-SY5Y cell line stably overexpressing transgenic SP-htau we demonstrated its secretion to the culture medium. Notably, treatment of this medium with PNGase-F to remove N-glycans enhanced aggregation propensity compared to untreated medium, while medium from control cells not expressing SP-htau showed hardly any aggregation whether treated or untreated with this enzyme. This suggests that the differences in aggregation levels are primarily due to N-glycosylation of SP-htau itself. N-glycosylated SP-htau is less aggregative possibly due to the presence of the bulky glycan molecules at all or some of the three putative N-glycosylation sites in htau (N167, N359, N410) which makes the protein sterically bulky and hampers the likelyhood that two htau monomers will be close enough to each other to allow self assembly and aggregation^39^. In accordance, the morphology of the aggregates formed in the sapmle containing SP-htau was transformed from thin filaments to wide bundles following PNGase-F treatment. Interestingly, a similar structure was reported when PHF from AD brain was treated with PNGase-F^13^. This indicates that the cell-based system described here is a valid model for studing the effect of N-glycosylation on htau aggregation in AD and its mechanism.

In conclusion, the current results are in agreement with previous reports regarding the possible effect of N-glycosylation on protein structure suggesting that the decoration of proteins with N-glycans preserves their solubility. The effect of N-glycans may depend on the nature of carbohydrate units attached to the amino acids, their position and the residues which surround the consensus sequence for N-glycosylation^17,30^.

## Materials and Methods

### Construction of pcDNA4 vector carrying htau fused to a signal peptide

The sequence encoding signal peptide originated by PCR from pCMV/myc/ER (V820-23, Thermo Fisher Scientific), using the following primers: F: 5′-GGCCGCGAATTCATGGGATGGAGCTGTATCATCCTC and R: 5′-CTGGCGGGGCTCAGCCATGGAGTGCGCGCCTGTGGA (the bold sequence represents restriction site of *Eco*R1; the single-underlined sequence represents part of the signal peptide; the double-underlined sequence contains part of the sequence of the longest htau isoform).

The htau-encoding sequence was amplified from the pNG2 plasmid (courtesy of Prof. Eckhard Mandelkow, DZNE, Bonn, Germany)^40^, using the following primers: F: product of the above mentioned PCR, and R: 5′-GC**CTCGAG**TCACAAACCCTGCTTGGCCAGG (the bold sequence represents *Xho*1 restriction site; the double-underlined sequence contains part of htau sequence. These two PCR reactions produced a sequence of signal peptide, followed by a wild type sequence of htau. Finally, the construct was cloned in to a pcDNA4 mammalian expression vector (Invitrogen) using the *Eco*R1 and *Xho*1 restriction sites. The sequence of the construct, termed hereafter SP-htau, was verified prior using.

### Maintenance of cultured cells and stable transfection

SH-SY5Y (ATCC CRL-2266) cells were cultured in Dulbecco’s Modified Eagle’s Medium /Nutrient Mixture F-12 (DMEM-F12) (Biological Industries) supplemented with 10% fetal bovine serum (Biological Industries), 1% L-glutamine (Biological Industries), 1% Pen-Strep Nystain (Biological Industries), and 1% non-essential amino acids (Biological Industries). Cells were incubated under 5% CO_2_ at 37 °C. Cells were stably transfected with the pcDNA4 plasmid containing SP-htau, using Lipofectamine LTX (Invitrogen). Cells were subcloned and one colony was chosen for subsequent experiments.

For selection, Zeocin antibiotic (Tamar laboratories) at a concentration of 150 μg/mL was added from day three post transfection throughout the culture period.

### Immunofluorescence staining

Monolayer culture cells grown on cover slips coated with Poly-D-Lysine (EMD Millipore) were fixed for 15 min in 4% paraformaldehyde (PFA) in phosphate buffered saline (PBS) and washed three times for 5 min each in PBS. The cells were incubated with 0.25% Triton X-100 in PBS for 10 min at room temperature, followed by three times washing in PBS, 5 min each. The cells were then blocked for 30 min in blocking solution (1% BSA, diluted in PBS, 0.1% Tween-20 (PBS-T)) and incubated overnight at 4 °C with blocking solution which contained primary antibody recognizing total tau (5A6, Hybridoma bank, 1:200) and primary antibody recognizing Calnexin (sc-11397, Santa Cruz Biotechnology, 1:200). On the next day, the cells were washed three times in PBS, 5 min each, followed by 2 h of incubation with the secondary antibodies: goat Anti-Mouse IgG Cy3 (115-165-003, Jackson ImmunoResearch, 1:200) and goat Anti-Rabbit IgG Cy5 (sc-45101, Santa Cruz Biotechnology, 1:200). Finally, cells were washed three times for 5 min each in PBS and mounted using VECTASHILED medium containing DAPI (H-1200, Vector Laboratories). Images were taken with LSM510 confocal microscope (Zeiss).

We performed immunofluorescence staining with an additional ER marker against GRP78/BIP, using the following protocol: The cells grown on cover slips coated with Poly-D-Lysine (EMD Millipore) were fixed for 5 min with 100% Methanol at −20 °C and additional 5 min with Methanol:Acetone (1:1) at −20 °C, followed by two washes in PBS 5 min each and one wash with 2% PBS/BSA. The cells then were blocked for 30 min in blocking solution (normal goat IgG in 2% PBS/BSA 1:100), washed with 0.5 ml of 2% PBS/BSA and incubated with primary antibody recognizing total tau (5A6, Hybridoma bank, 1:200) together with primary antibody recognizing GRP78/BIP (G9043, Sigma-Aldrich, 1:200) for 2 h. Next, cells were washed three times with 2% PBS/BSA followed by 1 h incubation with the secondary antibodies: goat Anti-Mouse IgG Cy3 (ab97035, 1:100, abcam) and goat Anti-Rabbit IgG Cy5 (111-175-144, Jackson ImmunoResearch, 1:100). Finally, cells were washed three times for 5 min each in PBS and mounted using VECTASHILED medium containing DAPI (H-1200, Vector Laboratories). Images were taken with LSM510 confocal microscope (Zeiss).

### Sample preparation for Western blot and aggregation analysis

Cells (3 × 10^6^) from each cell line (SH-SY5Y and SP-htau expressing SH-SY5Y) were plated in growth medium and incubated in a 5% CO_2_ at 37 °C for 24 h. Then the medium was replaced with fresh same medium lacking fetal bovine serum and incubation was continued for 60 h under 5% CO_2_ at 37 °C. Next, the media from the various cell lines were collected separately and centrifuged at 700 rpm to remove dead cells. The supernatant was collected and concentrated from 10 mL to 100 µL using centrifugal filters (50 kDa, Amicon Ultra, Merck). The concentrated media were then used for Western blotting or diluted with PBS pH 7.4 (Dulbecco’s phosphate buffer saline) for biophysical studies.

### PNGase-F treatment

The concentrated media, collected from SH-SY5Y and SP-htau expressing SH-SY5Y were treated with the glycosidase, peptide-N4-(N-acetyl-beta-glucosaminyl) asparagine amidase (PNGase-F, Sigma-Aldrich) to cleave off N-linked glycosidic linkages, according to manufacturer’s protocol with few modifications. Each concentrated medium (100 µL) was first treated with 3.4 µL Na_2_HPO_4_ (1 M), and 1.0 µL of NaH_2_PO_4_ (1 M). Then the samples were boiled for 10 min at 100 °C, allowed to cool down in ice bath and 3.5 units of PNGase-F were added. The samples were incubated for 3 h at 37 °C, followed by centrifugation using centrifugal filters (50 kDa, Amicon Ultra, Merck) in an attempt to remove the PNGase-F, excess salt and the detached glycans. Removal of glycans from the SP-htau protein was verified by Western blot analysis.

### Dot blot analysis

Cells (2 × 10^6^) from each cell line (SH-SY5Y and SP-htau expressing SH-SY5Y) were plated in growth medium and incubated under 5% CO_2_ at 37 °C for 24 h. Then the medium was replaced with fresh same medium lacking fetal bovine serum and incubation continued for 60 h in a 5% CO_2_ at 37 °C. Next the media from the various cell lines were collected separately and centrifuged at 5000 rpm to remove dead cells. The supernatant was collected and concentrated from 6 mL to 500 µL using centrifugal filters (50 kDa, Sartorius). The concentrated media were transferred to PVDF membrane (GE healthcare) using a Bio-Dot apparatus (Bio-Rad). The membrane was subsequently fixed in 4% paraformaldehyde (PFA), washed two times for 5 min each in Tris Buffered Saline (TBS), 0.1% Tween-20 (TBS-T), blocked for 1 h in blocking solution (5% milk, diluted in TBS-T) and incubated overnight at 4 °C with primary antibody (5A6, Hybridoma Bank, 1:1000) recognizing total tau, diluted in blocking solution. Next, the membrane was washed three times for 5 min each in TBS-T, incubated 1 h with goat anti mouse secondary antibody (sc-2060, Santa Cruz Biotechnology, 1:10,000) and washed again three times for 5 min each in TBS-T. The membrane was developed using Luminata Forte Western HRP Substrate (Millipore), according to the manufacturer’s instructions, and developed using Amersham Imager 600 (GE healthcare).

### Western blot analysis for total cellular proteins

The same cells (SH-SY5Y and SP-htau expressing SH-SY5Y) which were used for Dot blot analysis, were harvested and lysed, immediately after the media was removed, in 1 mL extraction buffer (20 mM Tris-HCl, pH 7.5, 100 mM NaCl, 5 mM EDTA, 1 mM MgCl_2_, 0.5% NP-40) containing a protease inhibitor cocktail (Roche) and 100 mM phenylmethanesulfonyl fluoride (PMSF). Next, lysates were incubated on ice for 30 min then were centrifuged at 10,300 rpm for 30 min at 4 °C. The supernatants were collected and boiled at 100 °C for 10 min in sample buffer containing β-mercaptoethanol. Samples were resolved in 4–20% (w/v) polyacrylamide gels (GenScript) using a Mini-PROTEAN Tetra Vertical Electrophoresis Cell apparatus (Bio-Rad), and were transferred onto polyvinylidene difluoride (PVDF) membrane by a semi-dry blot technique using eBlot Protein Transfer Device (GenScript). Next, the membrane was blocked for 1 h in blocking solution (5% milk, diluted in Tris Buffered Saline (TBS), 0.1% Tween-20 (TBS-T)) and incubated overnight at 4 °C with the primary antibody recognizing actin (ab8224, abcam, 1:10,000) diluted in blocking solution. On the following day, the membrane was washed three times for 5 min each in TBS-T and incubated 1 h with goat anti mouse secondary antibody (sc-2060, Santa Cruz Biotechnology, 1:10,000). The membrane was developed using Luminata Crescendo Western HRP Substrate (Millipore), according to the manufacturer’s instructions, and developed using Amersham Imager 600 (GE healthcare).

### Western blot analysis for secreted htau

The proteins in concentrated media (see PNGase-F treatment section) were resolved in 4–20% (w/v) polyacrylamide gels (GenScript) and were transferred onto PVDF membrane, as described in the previous section. The membrane was then blocked for 1 h in blocking solution (5% milk, diluted in Tris Buffered Saline (TBS), 0.1% Tween-20 (TBS-T)) and incubated overnight at 4 °C with the primary antibody recognizing total tau (5A6, Hybridoma Bank, 1:1000) diluted in blocking solution. Next day, the membrane was washed three times for 10 min each in TBS-T and incubated 1 h with goat anti mouse secondary antibody (sc-2060, Santa Cruz Biotechnology, 1:10,000). The membrane was developed using EZ-ECL (Biological Industries), according to the manufacturer’s instructions, and exposed to Fuji Medical X-Ray Film which were developed using Kodak X-OMAT.

### Mass spectrometry

For identification, tau digest peptides were loaded onto a bespoke column (15 cm 75 mm, fused silica) packed with beads (Jupiter C-18, 300 mm, 5 mm; Phenomenex, Torrance, CA) and connected to an Ekspert nano LC system (Eksigent, Dublin, CA). Elution was performed with Buffer A (acetonitrile (2%) with formic acid (0.1%)) and Buffer B (acetonitrile (80%) with formic acid (0.1%)), with a linear gradient (5–65% Buffer B, 45 min). MS peptide analysis and tandem MS fragmentation were performed with an LTQ-Orbitrap spectrometer (Thermo Scientific), operated in the data-dependent mode to enable switching between MS and collision-induced dissociation tandem MS analyses of the top five ions. Collision-induced dissociation fragmentation was performed at 35% collision energy with a 30 ms activation time.

Tau was identified and validated by using the SEQUEST algorithm in Proteome Discoverer software (Thermo Scientific) and the Uniprot- Swissprot FASTA database. Mass tolerance for precursor and fragmentations were set to 10 ppm and 0.8 Da, respectively. Only high-confidence peptides with the best XCorr score as obtained by the standard Percolator node parameters were chosen.

### Thioflavin T/S fluorescence assay

Stock solutions of Thioflavin T or Thioflavin S (ThT/ThS, 200 µM, Sigma-Aldrich) and heparin (100 µM, Sigma-Aldrich) were prepared in PBS (pH 7.4). For ThT/ThS experiments, the stock solution was diluted to 200 µL in each well of a 96-well black plate so that the final mixture contained 80 µL of the concentrated medium (untreated or PNGase-F treated) and 100 µL of ThT/ThS. Prior to the experiment, heparin was added (20 µL in each well) to initiate protein aggregation. Kinetic fluorescence data were collected for 5610 min at 37 °C in triplicate using Infinite M200 microplate fluorescence reader (Tecan, Switzerland), with measurements taken at 15 min intervals. Excitation and emission wavelengths were 440 nm and 490 nm, respectively. All of the experiments were repeated 3–4 times to ensure reproducibility.

### Transmission electron microscopy (TEM)

Aliquots (10 μL) from the aggregated concentrated media were applied onto the dark side of 400-mesh copper grids covered with carbon-stabilized Formvar film (Electron Microscopy Sciences) and allowed to float for 2 min. Excess solution was removed using blotting paper. Then, 2% uranyl acetate solution (10 μL) was added to the grid and allowed to float for 2 min. Excess solution was removed using blotting paper. The grid was dried at room temperature and was kept in a desiccator before taking TEM analysis on JEOL (Model: JEM 1400) instrument at 80 kV.

### Congo red birefringence

Aliquots (10 μL) from PNGase F-treated or untreated aggregated samples was placed over a glass slide followed by 10 μL of the saturated Congo red solution (in 80% aqueous ethanol). Excess solution was removed using a blotting paper. The sample was dried at room temperature and analyzed under a Nikon HD polarizable microscope under cross polarized light.

### Immuno-Gold Assay

A 2 μL aliquot from the PNGase F-treated or untreated aggregated concentrated media was applied onto the dark side of 400-mesh copper grid covered with carbon-stabilized Formvar film (Electron Microscopy Sciences) and allowed to float for 2 min. Excess solution was removed using blotting paper and the grid was allowed to dry for 2 min^28^. Then, the grid was blocked with SuperBlock blocking buffer (Thermo Scientific) for 30 min. Samples were incubated with the primary antibody recognizing total tau (ab64193, abcam, 1:100) in blocking buffer for 30 min, washed five times with the same buffer solution, and then incubated with secondary goat anti-rabbit antibody conjugated with 18-nm gold (111-215-144, Jackson ImmunoResearch, 1:20) for 30 min and similarly washed. Samples were viewed using a JEM-1400Plus electron microscope operating at 80 kV.

### ANS fluorescence assay

A 20 μL aliquot from the PNGase F-treated or untreated aggregated concentrated media was mixed with 100 μM ANS in PBS (Sigma-Aldrich). ANS fluorescence intensities were measured with excitation at 380 nm and emission between 400 and 750 nm using Infinite M200 microplate fluorescence reader (Tecan, Switzerland).

### Life/Dead cell counting

Cells (1 × 10^6^) from each cell line (SH-SY5Y and SP-htau expressing SH-SY5Y) were plated in growth medium and incubated in a 5% CO_2_ at 37 °C for 24 h. Then the medium was replaced with fresh same medium FBS and incubation was continued for 60 h under 5% CO_2_ at 37 °C. For the control experiment, the medium was replaced with fresh regular media with FBS and incubated in a similar manner. After 60 h, the media from the various cell lines were collected separately and mixed with the respective trypsinized cells followed by centrifugation at 700 rpm. Then the supernatant was discarded and 1 mL of regular media was added to each of the cell pellets and total number of cell vs percentage of live/dead cells were counted using Countess® II Automated Cell Counter from Thermo Fisher Scientific.

### Fluorescent live/dead staining assay

Cells (1 × 10^5^) from each cell line (SH-SY5Y and SP-htau expressing SH-SY5Y) were seeded in 24 well plate in growth medium and incubated in a 5% CO_2_ at 37 °C for 24 h. Then the medium was replaced with fresh same medium lacking FBS and incubation was continued for 60 h under 5% CO_2_ at 37 °C. For the control experiment, the medium was replaced with fresh regular media with FBS and incubated in a similar manner. After 60 h, we performed fluorescent live/dead staining assay (SigmaAldrich) containing fluorescein diacetate (6.6 μg/mL) and propidium iodide (5 μg/mL) to visualize the proportion of viable versus nonviable cells. The dye solutions were added directly to the cells containing no FBS. Whereas, for control experiment, the FBS containing media was replaced by fresh media containing no FBS followed by addition of dye solution. Then, the stained cells were immediately viewed under Nikon Eclipse Tifluorescent microscope with ZylascMOS camera using Nikon Intensilight C-HGFI fluorescent lamp.

## Supplementary information

Supplementary Information

## Data Availability

The datasets generated and analyzed during the current study are available from the corresponding author upon reasonable request.
