# Supplementary material for: Novel model of secreted human tau protein reveals the impact of the abnormal N-glycosylation of tau on its aggregation propensity
Source: Sci Rep. 2019 Feb 19;9:2254. doi: 10.1038/s41598-019-39218-x (PMC6381127; doi:10.1038/s41598-019-39218-x)
Supplement: Supplementary file 1 — Supplementary Information [file 41598_2019_39218_MOESM1_ESM.docx]

**Supplementary Information**

**Novel model of secreted human tau protein reveals the impact of the abnormal N-glycosylation of tau on its aggregation propensity**

*Yelena Losev, ^#1,^ Ashim Paul, ^#1^* *Moran Frenkel-Pinter,^1^ Malak Abu-Hussein,^1^ Isam Khalaila,^2^ Ehud Gazit^1,3^ & Daniel Segal^1,4^**

#Authors equally contributed


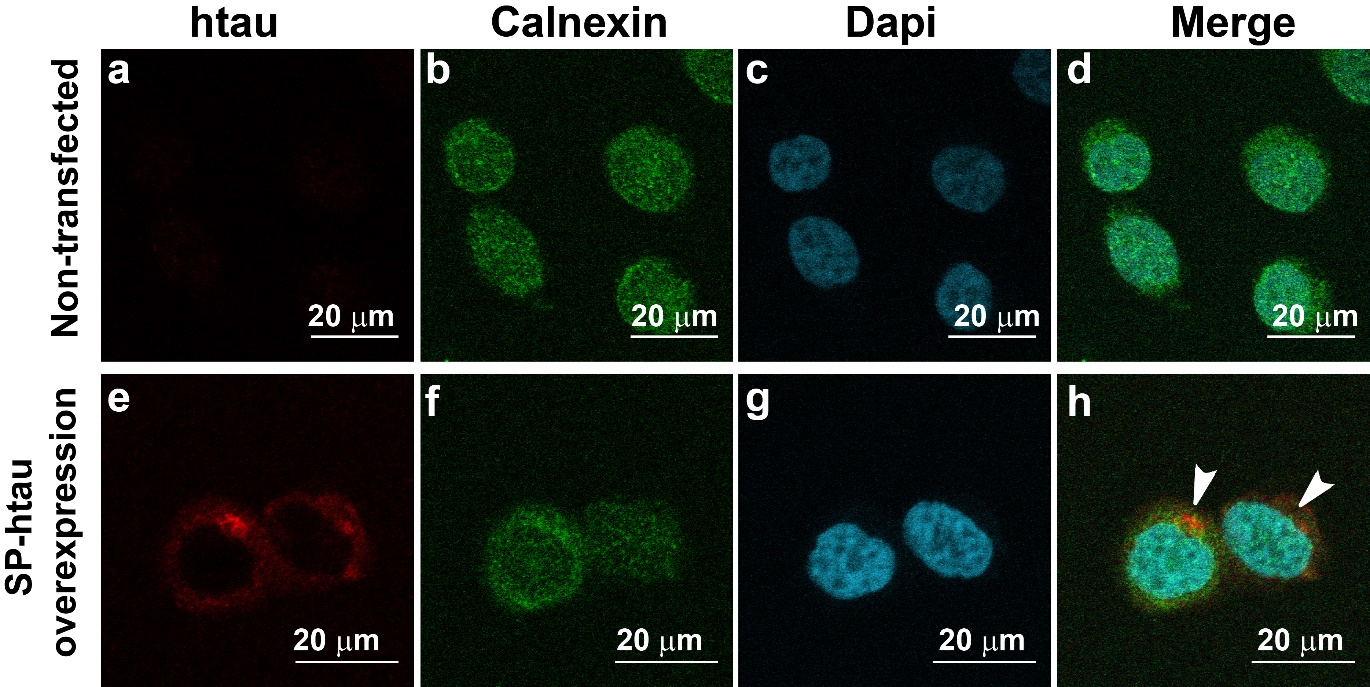


**Supplementary Fig. S1:** ***SP-htau overexpressed in SH-SY5Y is colocalized with Calnexin to the ER* (zoom images).** (a-d) Non-transfected SH-SY5Y cells; (e-h) SH-SY5Y cells stably transfected with SP-htau; (a, e) htau staining using 5A6 antibody against total tau; (b, f) anti Calnexin staining; (c, g) nuclear staining using DAPI; (d) Merge of a, b, c. (h) Merge of e, f, g. The arrows point to the colocalization between the tau and Calnexin.


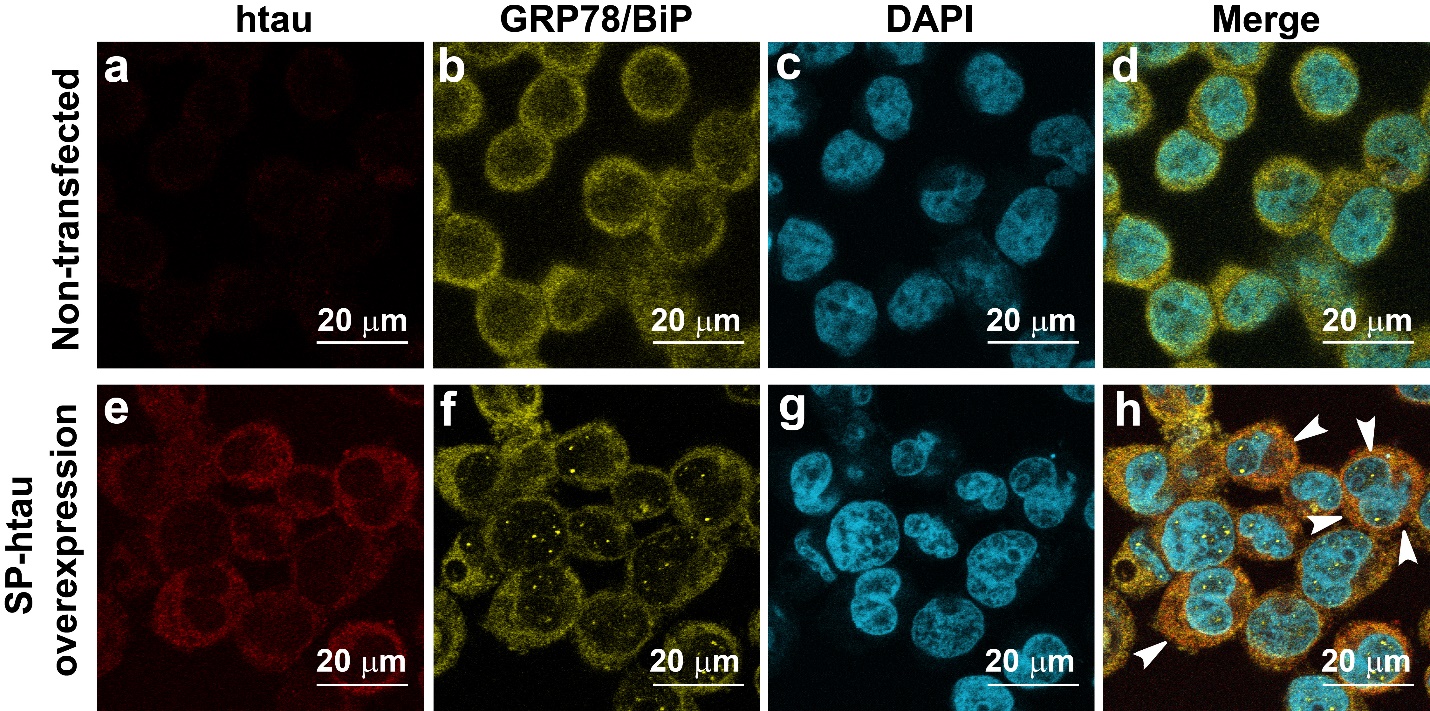


**Supplementary Fig. S2:** ***SP-htau overexpressed in SH-SY5Y is colocalized with GRP78/BiP to the ER* (zoom images).** (a-d) Non-transfected SH-SY5Y cells; (e-h) SH-SY5Y cells stably transfected with SP-htau; (a, e) htau staining using 5A6 antibody against total tau; (b, f) anti GRP78/BiP staining; (c, g) nuclear staining using DAPI; (d) Merge of a, b, c. (h) Merge of e, f, g. The arrows point to the colocalization between the tau and GRP78/BiP.


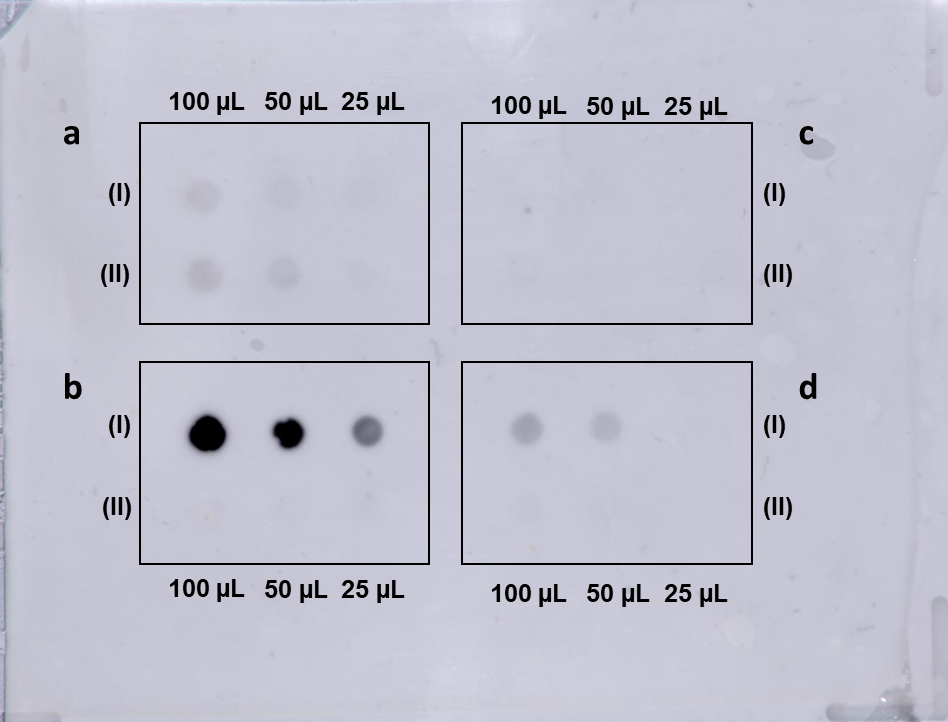


**Supplementary Fig. S3** ***Dot blot of the secreted SP-htau:*** Cells overexpressing SP-htau (a-d, I) and control non-transfected cells (a-d, II) were plated in growth medium containing FBS and incubated for 24 h. Then the medium was replaced either with fresh same medium (a,c) or medium lacking FBS (b,d) for additional 60h. Concentrated medium (a,b) and non-concentrated medium (c,d) were loaded on the PVDF membrane and dot blot was performed using 5A6 antibody (against total tau). Panel-b is shown as Fig.3a in the manuscript.

Note that when dot blot was performed in presence of FBS, but not in absence of FBS, we noticed that the medium could not pass through the membrane. Taking these observations into account we believe that the signal observed in presence of FBS (Supplementary Fig. S3a) may be due to non-specific reaction of the antibody with proteins present in the FBS. This may be a reason for a low signal we observed both in the control cell and cells overexpressing SP-htau.


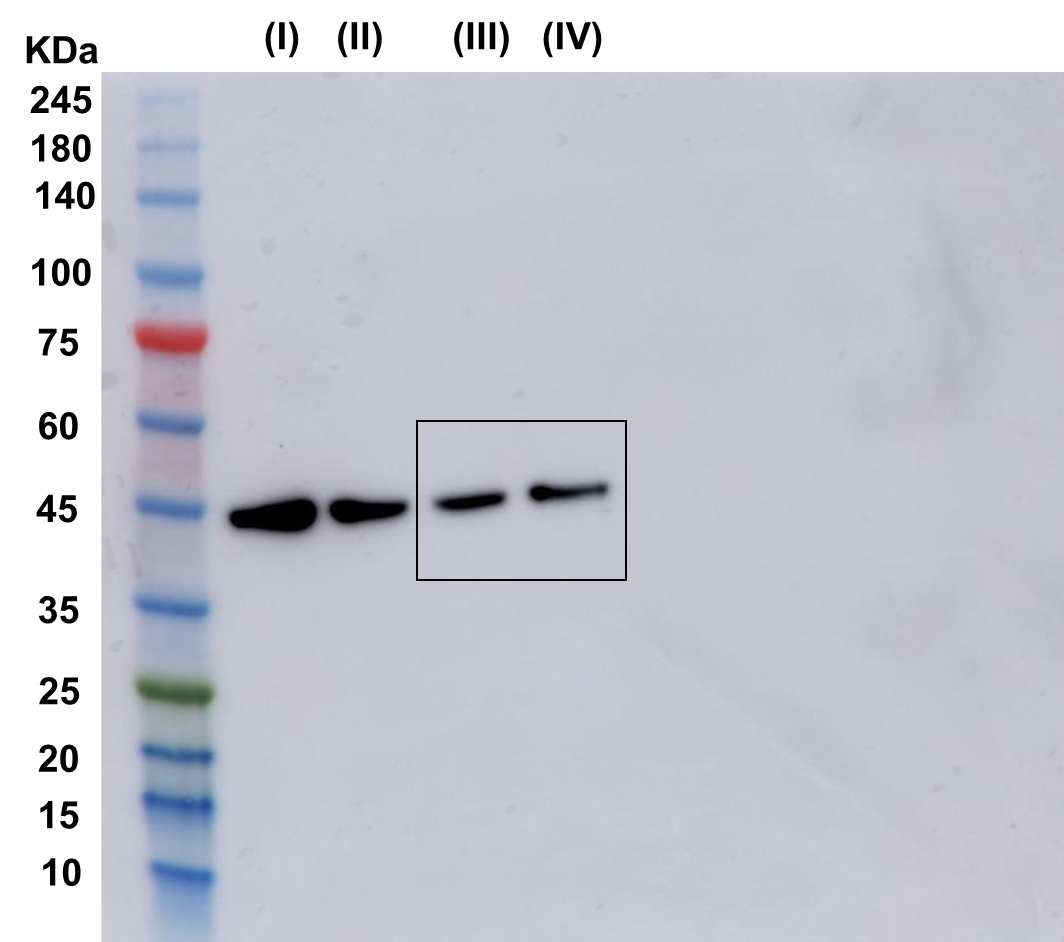


**Supplementary Fig. S4** ***Western blot analysis for evaluation of total cellular protein level using β-actin****:* Total protein level was evaluated by measuring actin level in the same cell lines used for dot blot analysis, overexpressing SP-htau (I and III) and control non-transfected cells (II and IV). The cells were incubated with FBS for 24 h and replaced either with fresh same medium (I and II) or medium lacking FBS (III and IV) and incubated for additional 60h. The total protein evaluation was performed only for the concentrated samples on Western blot, using ab8228 antibody (against β-actin). The demarcated section is used as Fig. 3b in the manuscript.

**Supplementary Table S1:** ***The impact of the presence or absence of FBS on the total number of cells and their viability***

| Cell systems | Total # of cells | Avg. % of live cells | Avg. % of dead cells |
| --- | --- | --- | --- |
| Control cells cultured with FBS | 1.47x10^7^ | 91 | 9 |
| Control cells cultured without FBS | 6.65x10^6^ | 84 | 16 |
| SP-htau overexpressing cells cultured with FBS | 1.25x10^7^ | 90 | 10 |
| SP-htau overexpressing cells cultured without FBS | 8.25x10^6^ | 85 | 15 |


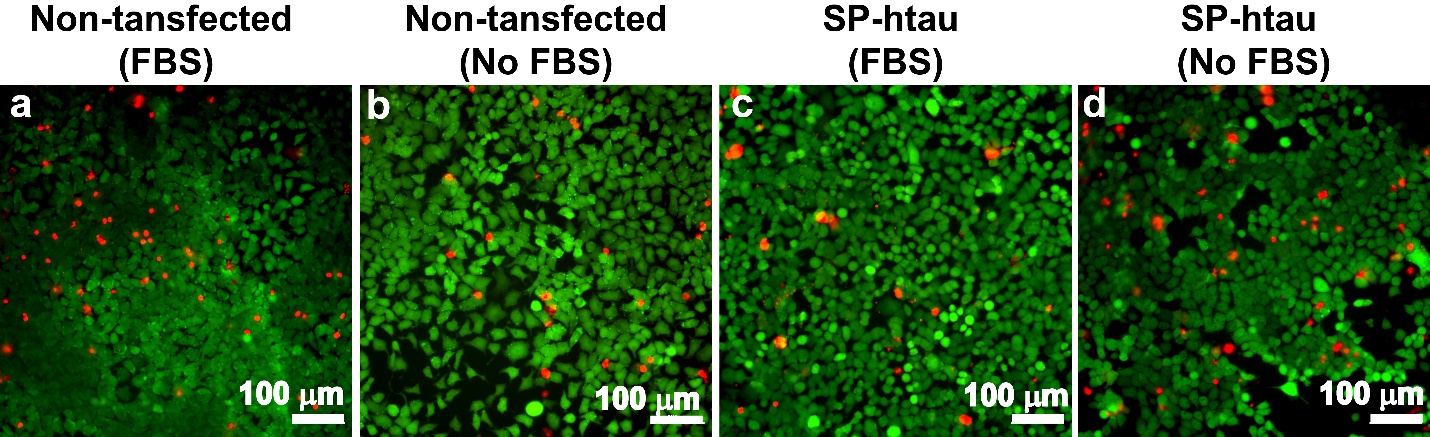


**Supplementary Fig. S5**: ***Fluorescence live/dead staining assay.*** (a,b) Non-transfected SH-SY5Y cells; (c,d) SH-SY5Y cells stably transfected with SP-htau. (a, c) in presence of FBS, (b, d) in absence of FBS.

**Supplementary Table S2:** ***Tryptic digest analysis of the immunoprecipitated tau***

**
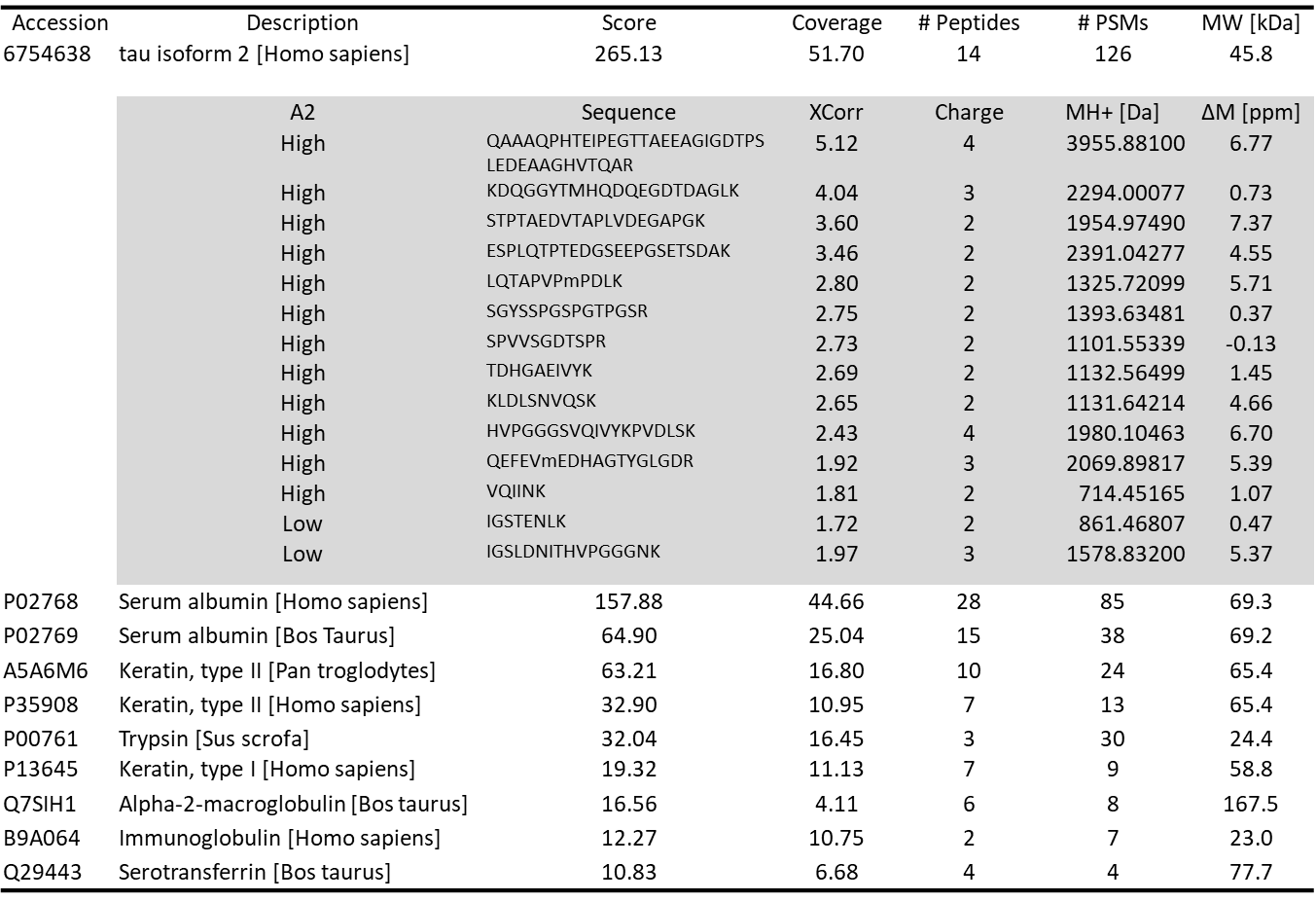
**


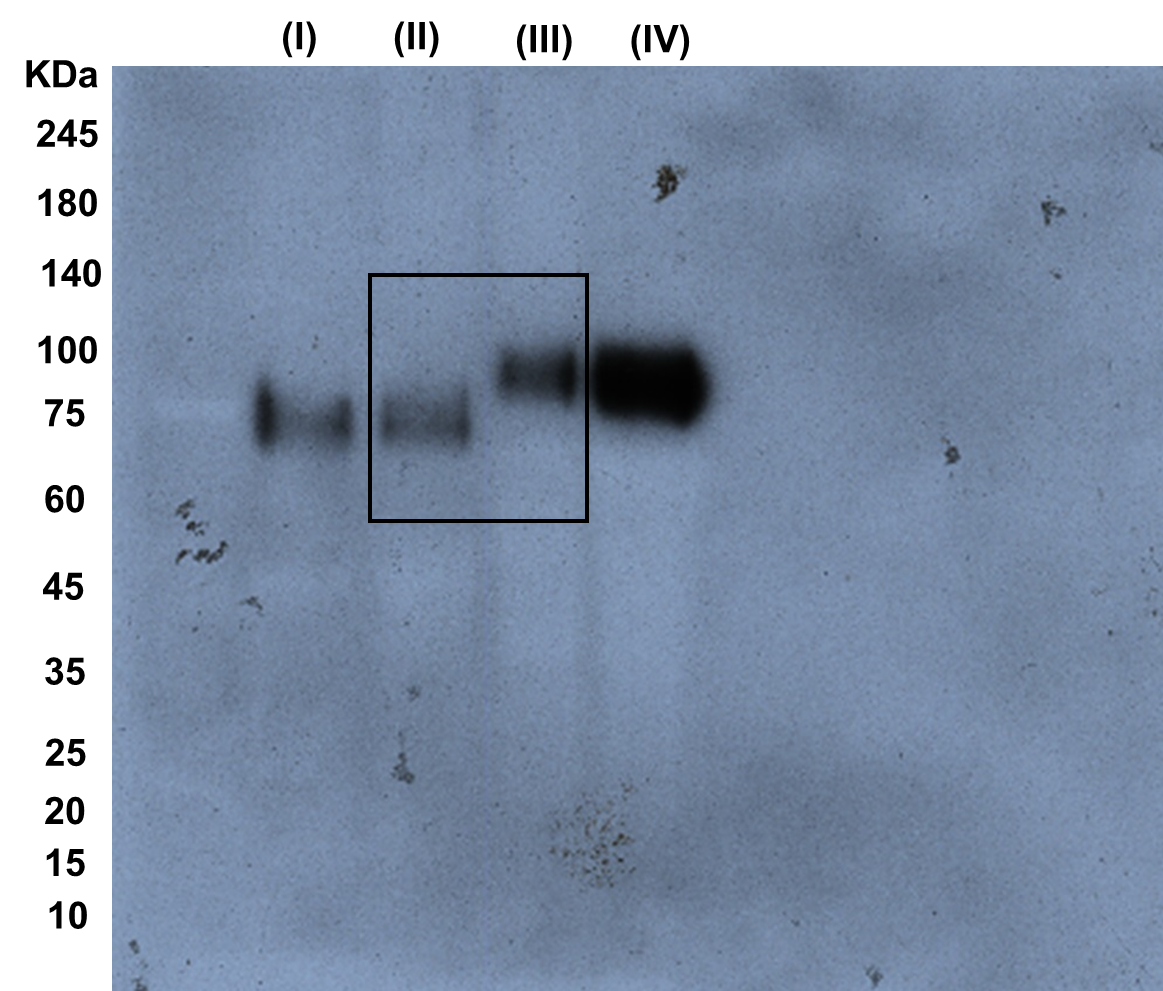


**Supplementary Fig. S6:** ***Western blot analysis of total tau in growth medium of SH-SY5Y cells overexpressing SP-htau treated or untreated with PNGase-F.*** (I and II) with enzyme treatment, (III and IV) without treatment. Sample I was treated according to the manufacturer’s protocol, samples II and III were treated as mentioned in the Material and Method section and sample IV was untreated. The demarcated section is used as Fig. 3c in the manuscript.


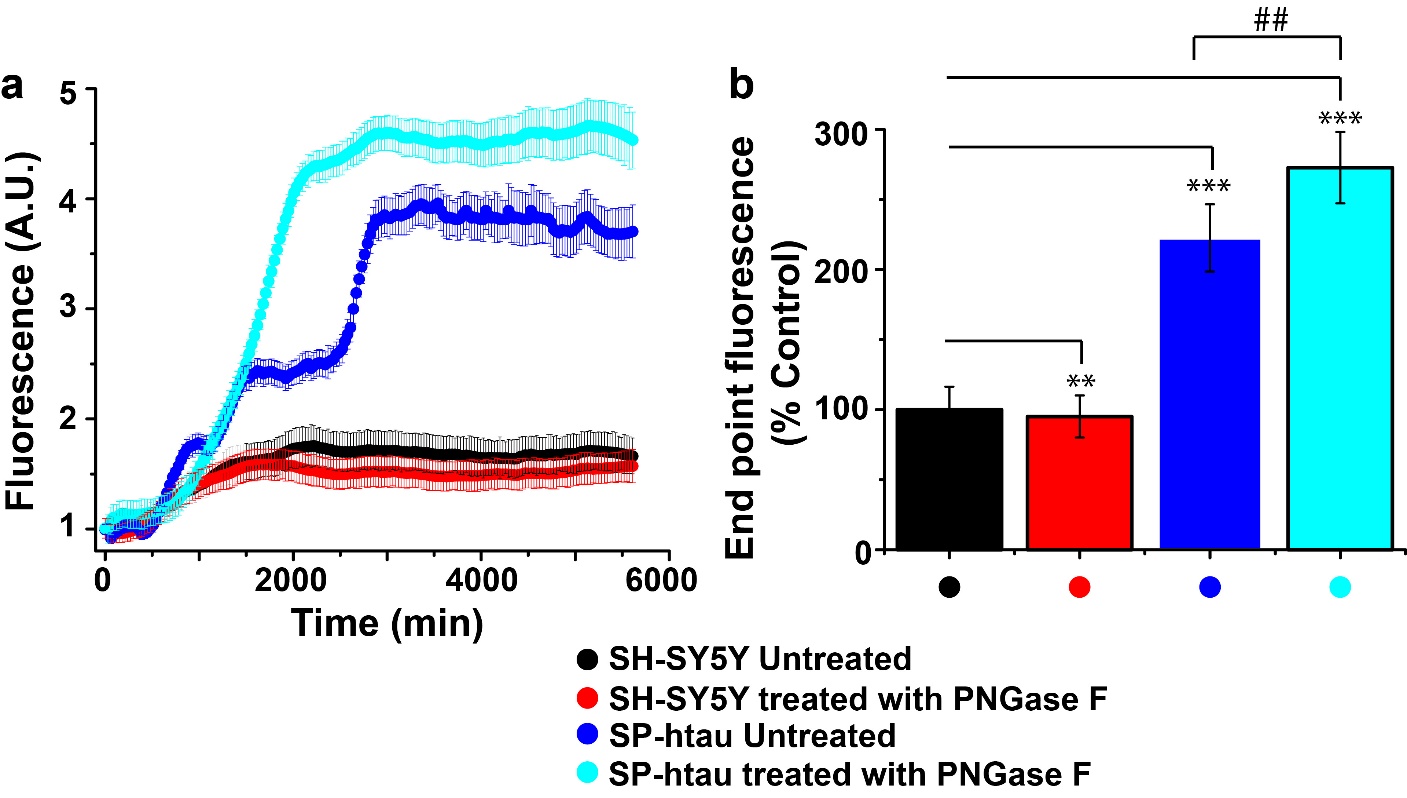


**Supplementary Fig. S7: *Time dependent Thioflavin-S binding kinetics of culture medium.*** (a) non-transfected SH-SY5Y cells treated (red) or untreated with PNGase-F (black), and of SH-SY5Y cells expressing SP-htau prior to (blue) and following PNGase-F treatment (cyan). (b) Relative ThS signal at the end point of incubation at 37 °C. Non-transfected untreated cells were set as 100%. P-values: **p<0.05 ***p<0.001 ##P<0.05.


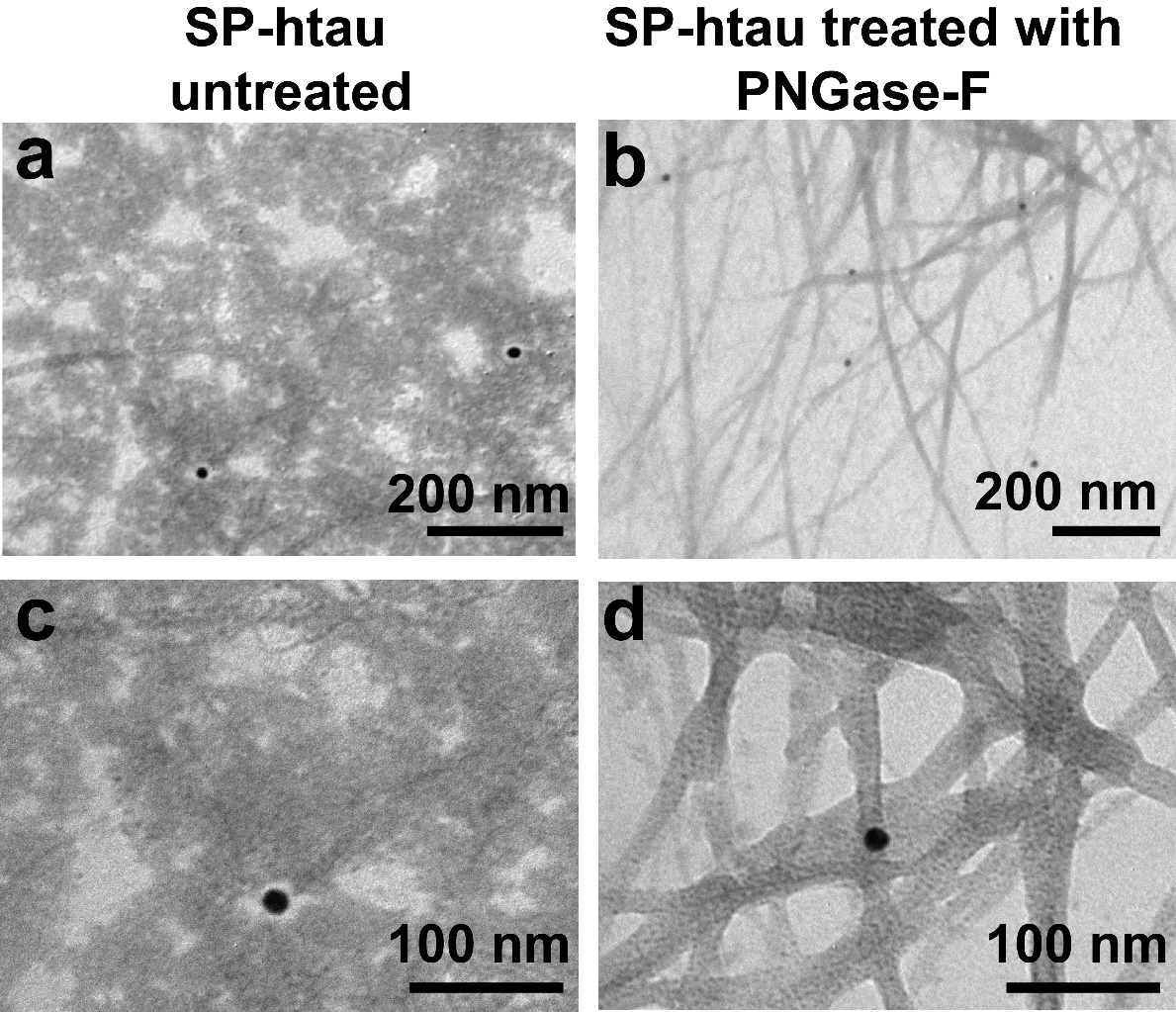


**Supplementary Fig. S8:** ***Immuno-gold labelling of aggregated SP-htau.*** (a,c) PNGase-F untreated sample and (b,d) PNGase-F treated sample. Antibody used ab64193 followed by goat anti-rabbit antibody conjugated with 18-nm gold.
